# Bilateral Traumatic Facial Paralysis with Hearing Impairment and Abducens Palsy

**DOI:** 10.1155/2020/8843187

**Published:** 2020-10-07

**Authors:** Imane Ouhbi, Taoufik Abdellaoui, Noureddine Errami, Fouad Benariba

**Affiliations:** ^1^Department of Otolaryngology, Military Hospital Mohamed V, Rabat, Morocco; ^2^Department of Ophthalmology, Military Hospital Mohamed V, Rabat, Morocco

## Abstract

The temporal bone is often affected in basilar skull fractures. Fractures involving the petrous portion are particularly significant, as they may be associated with neurovascular sequelae. Bilateral facial paralysis secondary to bilateral temporal bone fracture is a rare clinical entity, even more so when associated with other cranial nerve damage such as abducens nerve paralysis and hearing impairment. Only 4 similar cases have been reported in the literature to date. In this paper, we describe a 28-year-old male patient with bilateral facial paralysis, unilateral abducens palsy, and bilateral hearing loss due to bitemporal fractures that developed after a motor vehicle accident. Conservative management was preferred. The 6-month follow-up showed remarkable improvement. This report highlights the effectiveness of conservative management in posttraumatic complete facial and abducens palsy.

## 1. Introduction

The facial or abducens nerve damage following head injury is not uncommon. However, simultaneous sixth nerve and bilateral facial nerve palsies is extremely rare. Computed tomography (CT) is the best procedure for detecting the fracture line and for assessing any associated lesions within the temporal bone. The abducens nerve palsy usually recovers spontaneously, but there is controversy concerning the management of traumatic facial paralysis (FP). In this paper, we report a case of a young male who developed immediate bilateral facial and left abducens paralysis following a motor vehicle accident, which was managed conservatively with gratifying results.

## 2. Case Report

**Figure 1:**
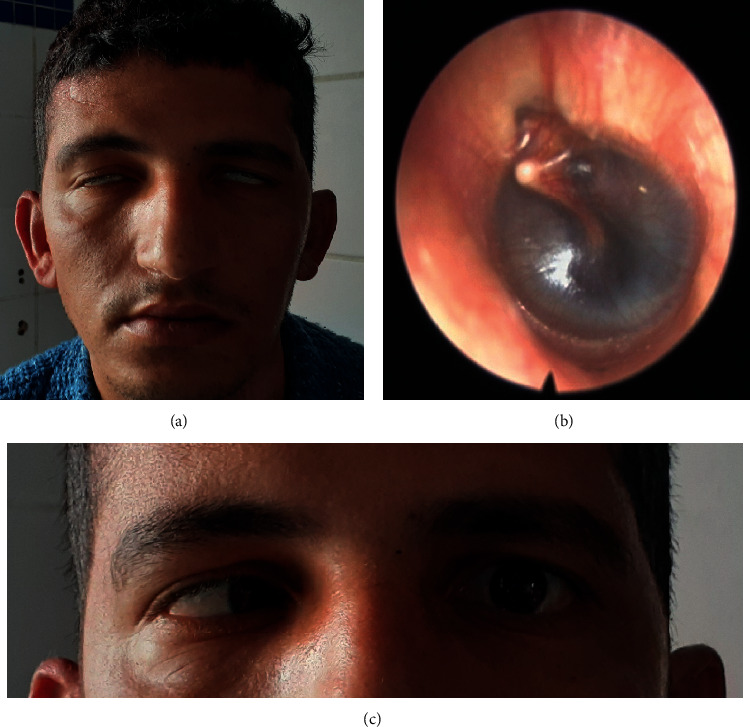
(a) Bilateral Charles Bell's phenomenon with mask-like face. (b) Left ear hemotympanum. (c) Left abducens palsy.

**Figure 2:**
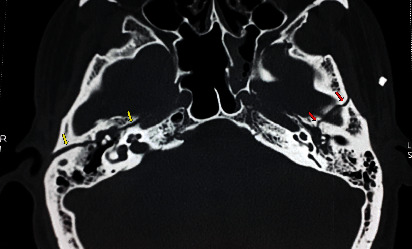
Transverse section of cranial CT (plane of incudomalleolar joint) showing right longitudinal otic-sparing temporal bone fracture (yellow arrows) and left transverse temporal bone fracture (red arrows).

A 28-year-old man was admitted after a motor vehicle accident with thoracic and craniofacial impacts. The patient was conscious, disoriented but cooperative. He was mildly dyspneic and complained of deafness and diplopia. The patient suffered thoracic contusions and a left hemothorax. On the otolaryngology level, the initial evaluation showed an expressionless and “mask-like face,” with barely perceptible mouth and frontal movements and incomplete eye closure, consistent with a bilateral grade 5 facial paralysis on the House–Brackmann grading scale (H–B). A bilateral Charles Bell's phenomenon was noted, as well as an abduction deficit on the left eye (Figures 1(a) and 1(c)). Otoscopy revealed bilateral hemotympanum (Figure 1(b)). The patient underwent a full audiological evaluation. The pure tone audiometry showed a right cophosis and a left mixed hearing loss. The tympanogram showed bilateral flat curves, and the stapedial reflex was abolished on both sides. An ophthalmological exam with a Lancaster test revealed a left abducens palsy. High-resolution computed tomography (HRCT) showed an otic-sparing longitudinal right temporal bone fractures and a transversal left temporal bone fracture (Figure 2).

The evolution was marked by the degradation of the respiratory functions which required management in an intensive care unit. No surgery was conceivable given the instability of his hemodynamic and respiratory state. Consensus was therefore reached to proceed with conservative management. Treatment regarding CN palsies was coordinated in conjunction with the otolaryngology and ophthalmology services. The patient had a short course of intravenous corticosteroids, with antibiotics and mucolytics for the hemotympanum. Passive facial physiotherapy was started as soon as possible. The ophthalmologist prescribed corneal protection.

**Figure 3:**
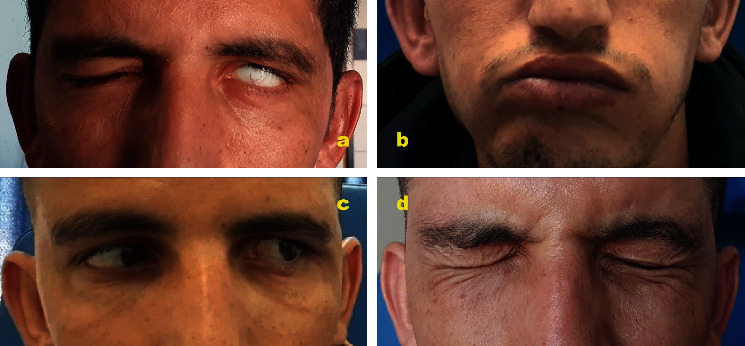
(a) Two-week follow-up: improvement of the right-side facial palsy. (b) Two-month follow-up: development of the mouth movement exhibiting the progressive return of function of the lower branches of the facial nerve. (c) Two-month follow-up: recovery of the left abducens palsy. (d) Six-month follow-up: evolution of the bilateral facial paralysis.

At the two-week follow-up, after the stabilization of the patient, we noticed a remarkable improvement concerning the right side of the facial palsy, becoming a grade 2 H–B (Figure 3)(a), which encouraged to pursue with the conservative approach. The hearing improved slowly and progressively as well, parallel to the resorption of the hemotympanum.

At the two-month follow-up, a slight return of function of the lower branches of the seventh nerve was noticed (Figure 3)(b), and the abducens palsy had spontaneously regressed (Figure 3)(c).

Six months after the accident, the patient exhibited H–B grade 1 recovery on the right and H–B grade 2 recovery on the left side (Figure 3)(d). The hearing on the other hand had improved partially. The audiometry showed a moderate right neurosensorial hearing loss (NSHL) and a mild left NSHL.

## 3. Discussion

Bilateral temporal bone fractures are uncommonly encountered. Posttraumatic bilateral facial palsy is an even rarer complication. The association of the sixth, seventh, and eight cranial nerves (CN) damage as it is in our case is exceptional. A review of the literature since 1961 revealed only 14 case reports about a bilateral traumatic facial paralysis. Only 4 of them were associated with other CN damages as well.

Fractures of the temporal bone can be categorized according to their relation to the long axis of the petrous segment: transverse (10% to 20%), longitudinal (80% to 90%), or a mixed pattern [1].

Conductive hearing loss is typical. Sensorineural hearing loss is often secondary to acoustic or concussive injury to the cochlea. Facial nerve paralysis is more commonly associated with transverse fractures than with longitudinal fractures (50% versus 15%, respectively) [2]. The incidence and morbidity is also greater with the transverse variety [3]. Facial paralysis after trauma can be immediate or delayed. Immediate onset is more common [2, 4]. The laceration, contusion, entrapment, crushing, or traction of the facial nerve at the site of fracture may cause the immediate onset of facial paralysis. Delayed onset usually suggests injury secondary to edema of the nerve with consequent compression of the nerve within its bony compartment [4].

On the other hand, the abducens nerve is less commonly injured. It is observed in 2.7% of patients with head injuries [5]. The long intracranial course of the nerve along with its rigid intracavernous attachment has been attributed to its vulnerability. The nerve may be impacted against the Gruber's (petrosphenoidal) ligament following an upward thrust or is injured when it impacts against the ridge of the petrous pyramid. The nerve trunk may also be contused at its entry point through the dural opening into Dorello's canal. [5, 6].

The early diagnosis of cranial nerves palsies in the traumatic brain injury patients can be particularly challenging due to the severe nature of the injury, especially when associated to cognitive deficits and other secondary complications. In addition, early detection of bilateral and symmetric facial nerve injury can be challenging because of the lack of facial asymmetry in associated movements. Thus, the detection of Bell's phenomenon and inadequate eyelid closure can be very helpful [7].

The reversibility of cranial nerve palsies depends on the type of nerve. The recovery of motor nerves in general is more satisfactory than that of sensorial nerves. In most instances, abducens palsies recover spontaneously, as in the case presented [8].

Management of CN injuries is mostly symptomatic. For sixth nerve palsy, occlusion of an eye with an eye-pad may be useful to alleviate the symptoms of diplopia. The use of Fresnel prism can also be helpful. Botulinum injection to weaken the medial rectus or strabismus surgery can also be considered [9]. The management of traumatic facial nerve injuries is controversial. Darrouzet et al. suggested that surgical decompression of the facial nerve is indicated when FP is total, is of immediate onset, and is associated with a bad prognosis electromyogram pattern with a clear-cut fracture line on the fallopian canal on HRCT. If the palsy is incomplete, delayed, or both, medical treatment by corticosteroids, physiotherapy, and rehabilitation could be considered [10]. Fortunately, in our case, medical treatment and early physiotherapy gave gratifying results.

## 4. Conclusion

Bilateral traumatic facial paralysis associated with abducens nerve palsy is an exceptional condition. Facial nerve recovery is the major concern, as abducens nerve palsy usually recovers spontaneously. This case report highlights the important of an early diagnosis and the efficiency of the conservative management.

## Data Availability

The data used to support the findings of this study are available from the corresponding author upon request.

## References

[1] Chan E. H., Tan H. M., Tan T. Y. (2005). Facial palsy from temporal bone lesions. *Annals of the Academy of Medicine, Singapore*.

[2] Davis R. E., Telischi F. F. (1995). Traumatic facial nerve injuries: review of diagnosis and treatment. *The Journal of Cranio-Maxillofacial Trauma*.

[3] Brodie H. A., Thompson T. C. (1997). Management of complications from 820 temporal bone fractures. *The American Journal of Otology*.

[4] Roth J., Toaff J. S., Margalit N., Salame K. (2007). Traumatic facial diplegia and horner syndrome: case report. *European Journal of Trauma and Emergency Surgery*.

[5] Lee G. Y. F., Halcrow S. (2002). Petrous to petrous fracture associated with bilateral abducens and facial nerve palsies: a case report. *The Journal of Trauma: Injury, Infection, and Critical Care*.

[6] Salunke P., Madhivanan K., Kamali N., Garg R. (2016). Spontaneous recovery of post-traumatic acute bilateral facial and abducens nerve palsy. *Asian Journal of Neurosurgery*.

[7] Undabeitia J., Liu B., Pendleton C., Nogues P., Noboa R., Undabeitia J. I. (2013). Bilateral traumatic facial paralysis. Case report. *Neurocirugía*.

[8] Holmes J. M., Droste P. J., Beck R. W. (1998). The natural history of acute traumatic sixth nerve palsy or paresis. *Journal of American Association for Pediatric Ophthalmology and Strabismus*.

[9] Azad T. D., Veeravagu A., Corrales C. E., Chow K. K., Fischbein N. J., Harris O. A. (2016). Abducens nerve avulsion and facial nerve palsy after temporal bone fracture: a rare concomitance of injuries. *World Neurosurgery*.

[10] Darrouzet V., Duclos J.-Y., Liguoro D., Truilhe Y., De Bonfils C., Bebear J.-P. (2001). Management of facial paralysis resulting from temporal bone fractures: our experience in 115 cases. *Otolaryngology-Head and Neck Surgery*.

